# Development of Loop-Mediated Isothermal Amplification Assays for Rapid and Sensitive Detection of Three Viruses Infecting *Areca catechu* L.

**DOI:** 10.3390/plants15203234

**Published:** 2026-10-21

**Authors:** Xiaoqing Niu, Yujing Tian, Jianguo Shen, Hui Zhu, Fangluan Gao

**Affiliations:** 1 Coconut Research Institute, Chinese Academy of Tropical Agricultural Sciences, Wenchang 571339, China; zhuhui@catas.cn; 2 Institute of Plant Virology, Fujian Agriculture and Forestry University, Fuzhou 350002, China; 3 Fuzhou Customs Technical Center, Fuzhou 350002, China

**Keywords:** YLD, APV1, APYLaOMV, APLTV1, RT-LAMP, optimal LAMP conditions

## Abstract

Yellow leaf disease (YLD) is a major threat to the areca palm (*Areca catechu* L.) industry. Accumulating evidence indicates that areca palm velarivirus 1 (APV1) is a key pathogen associated with YLD. In addition, areca palm latent totivirus 1 (APLTV1) and areca palm yellow leaf-associated ormycovirus (APYLaOMV) are two recently reported viruses that frequently co-infect areca palms. These three viruses can infect betel palms either individually or in combination, and their associated symptoms cannot be reliably distinguished by visual observation alone. In this study, through primer screening and systematic optimization of key factors affecting amplification efficiency, three independent one-step RT-LAMP assays were established for the detection of APV1, APLTV1, and APYLaOMV. The Mg^2+^ and dNTP concentrations, reaction temperatures, and inner-to-outer primer ratios were optimized to give detection limits of 10^−3^ for APYLaOMV, 10^−4^ for APLTV1, and 10^−5^ for APV1. The assays showed no cross-reactivity with the non-target areca palm viruses ANSSV and ANRSV, and were further validated on 26 field samples collected from Hainan Province, where their detection rates were comparable to or higher than those of conventional RT-PCR. Together, these results demonstrate that the developed RT-LAMP assays are sensitive and practical tools for routine molecular diagnosis and epidemiological investigation of APV1, APLTV1, and APYLaOMV in areca palms.

## 1. Introduction

The areca palm (*Areca catechu* L.) is an economically important cash crop, with large-scale cultivation distributed across South and Southeast Asia, the Pacific Islands, and parts of Africa [1]. However, areca palm production is severely threatened by yellow leaf disease (YLD), a destructive disorder first documented in India in 1914 [2]. Typical symptoms of YLD include gradual leaf chlorosis, bunchy top, and declining plant vitality, which result in drastic losses in both areca nut yield and quality [1,3]. Accumulating evidence confirms that areca palm velarivirus 1 (APV1) serves as the dominant pathogen responsible for YLD on Hainan Island, China [4,5,6,7,8]. This virus belongs to the genus *Velarivirus* (family *Closteroviridae*) and possesses a positive-sense single-stranded RNA ((+)ssRNA) genome with 11 open reading frames (ORFs). ORF1a encodes a large multifunctional polyprotein containing papain-like protease (P-Pro), methyltransferase (Met), and helicase (Hel) domains. ORF1b is translated through a +1 ribosomal frameshift from ORF1a and produces RNA-dependent RNA polymerase (RdRp). The subsequent ORFs code for a small hydrophobic protein (ORF2), heat shock protein 70 homolog (HSP70h, ORF3), a 64 kDa polypeptide (ORF4), and a protein of unknown function (ORF5). ORF6 and ORF7 encode the major coat protein (CP) and minor coat protein (CPm), respectively, whereas the functions of the proteins encoded by ORFs 8–10 have not been characterized [4].

Several additional viruses infecting areca palm have subsequently been identified, including areca palm necrotic ringspot virus (*Arepavirus arecae*, ANRSV) [9,10] and areca palm necrotic spindle-spot virus (*Arepavirus arecamaculatum*, ANSSV) [11], both of which are members of the genus *Arepavirus* (family *Potyviridae*) with +ssRNA genomes; areca palm latent totivirus 1 (APLTV1) [12]; and areca palm yellow leaf-associated ormycovirus (APYLaOMV) [13]. tobacco streak virus (TSV; genus *Ilarvirus*, family *Bromoviridae*; tripartite (+)ssRNA genome) [14]; APLTV1 is a member of the genus *Totivirus*, family *Totiviridae*, and possesses a non-segmented double-stranded RNA (dsRNA) genome that contains two ORFs, whose encoded proteins share 55% and 69% amino-acid sequence identity with the capsid protein and the RdRp of other totiviruses, respectively [12]. APYLaOMV is a putative member of the proposed genus *Alphaormycovirus*. Its genome is monopartite, spans 3063 nucleotides (excluding the 3′ poly(A) tail) and contains a single ORF that encodes a putative RdRp of 997 amino acid residues with the typical A-B-C motifs, in which motif C carries an NDD rather than the conventional GDD catalytic triad [13]. It should be noted that all previously known hosts of ormycoviruses are fungi, and APYLaOMV was the first ormycovirus-like sequence identified in areca palm: it was detected in YLD-affected leaves but not in healthy leaves [13], suggesting a close association with the yellowing disease.

To begin this study, we collected 544 areca palm yellowing leaf samples from 18 cities and counties across Hainan Province in 2023. Preliminary RT-PCR screening of these samples showed that APV1, APLTV1, and APYLaOMV were the three most frequently detected viruses, with average detection rates of 70.77%, 29.23%, and 33.64%, respectively [13]. These viruses may occur as single or mixed infections in areca palms, substantially reducing yield, nut quality, and the commercial value of the crop. Among the 544 areca palm leaf samples, the co-infection rates were 6.62% for APYLaOMV and APV1, 8.64% for APV1 and APLTV1, and 9.19% for APLaOMV and APLTV1, with 4.41% of the samples co-infected by all three viruses. Notably, no distinct symptomatic differences were observed among areca palms infected with these three viruses. In particular, infection by APLTV1 does not induce apparent phenotypic alteration in areca palms. Furthermore, these viruses can be occasionally detected in asymptomatic leaves, in line with the typical latent infection characteristic of plant viruses. The interaction mechanisms among the three viruses remain uncharacterized to date. As the disease progresses, plant growth is markedly inhibited, newly emerging leaves remain underdeveloped, and individual leaves become shorter.

Because the causal agents cannot be distinguished by symptoms alone, accurate laboratory diagnosis is required for disease surveillance and seedling quarantine. The detection of these viruses in areca palm mainly relies on enzyme-linked immunosorbent assay (ELISA) and reverse transcription polymerase chain reaction (RT-PCR) techniques [15,16]. While these conventional approaches offer reliable diagnostic performance, they generally require specialized laboratory equipment (e.g., thermal cyclers, real-time PCR instruments), sophisticated cold-chain logistics for sample transport, and trained technical personnel. These requirements restrict the capacity for timely, effective on-site detection of plant viruses, particularly in resource-limited agricultural settings.

Benefiting from the rapid advances in molecular diagnostic technology, various isothermal amplification strategies have been established for field testing. Among them, loop-mediated isothermal amplification (LAMP) has emerged as a well-established detection system [17]. This method employs four to six specifically designed primers to ensure outstanding detection specificity. Incubation in a simple water bath or heat block enables LAMP to generate approximately 10^3^-fold more amplicons within a short reaction period compared with conventional PCR, conferring remarkable advantages in time efficiency and cost control. Owing to such favorable properties, LAMP has been validated as a reliable and robust tool for the detection and identification of viral and microbial pathogens [17,18,19,20]. However, no RT-LAMP assay has yet been established for APV1, APLTV1 or APYLaOMV, leaving a diagnostic gap for the field surveillance of the major areca palm viruses.

Under field conditions, areca palms are frequently infected by multiple viruses, either singly or in combination, and the resulting symptoms often overlap or appear as nonspecific yellowing. Consequently, visual inspection alone cannot reliably identify the causal agent(s), and accurate laboratory diagnosis is required for disease surveillance and seedling quarantine. However, conventional RT-PCR depends on thermal cyclers, gel electrophoresis, and trained personnel, which limits its application in resource-limited settings such as plantations and nurseries. RT-LAMP overcomes these constraints, as it can be performed under isothermal conditions with minimal equipment, and it has been successfully applied to detect ANSSV and ANRSV [17], demonstrating its feasibility for areca palm viruses. Nevertheless, no RT-LAMP assay has yet been established for the three viruses APV1, APYLaOMV, and APLTV1, leaving a diagnostic gap for the simultaneous field surveillance of major areca palm viruses. To address this gap, in the present study we developed and evaluated RT-LAMP assays for APV1, APYLaOMV, and APLTV1. Conserved target regions were identified by multiple-sequence alignment of the complete APYLaOMV genome and the capsid protein (CP) gene sequences of APLTV1 and APV1 using DNAMAN 8. Following systematic optimization of key reaction parameters, these assays provide robust molecular tools for the rapid, sensitive, and field-deployable detection of the three viruses, with implications for virological research, disease surveillance, seedling quarantine, and integrated disease management.

## 2. Results

### 2.1. Screening of LAMP Primers for the Three Viruses

The aim of this step was to select, for each virus, the primer set giving the fastest and most efficient amplification. Total RNA was extracted from areca palm leaves that had previously been confirmed by RT-PCR and Sanger sequencing to be infected with APV1, APLTV1 or APYLaOMV, respectively, and first-strand cDNA was synthesized by reverse transcription (Section 4.2); the resulting cDNA was used as the template throughout. Each candidate primer set was tested in an otherwise identical reaction mixture (Section 4.4) incubated at 65 °C for 60 min. Reactions were monitored in real time by turbidimetry at 650 nm every 6 s, and, after incubation, SYBR Green I was added for visual colorimetric reading. A no-template control (NTC), in which the cDNA was replaced by an equal volume of RNase/DNase-free water, was included in every run.

**Figure 1:**
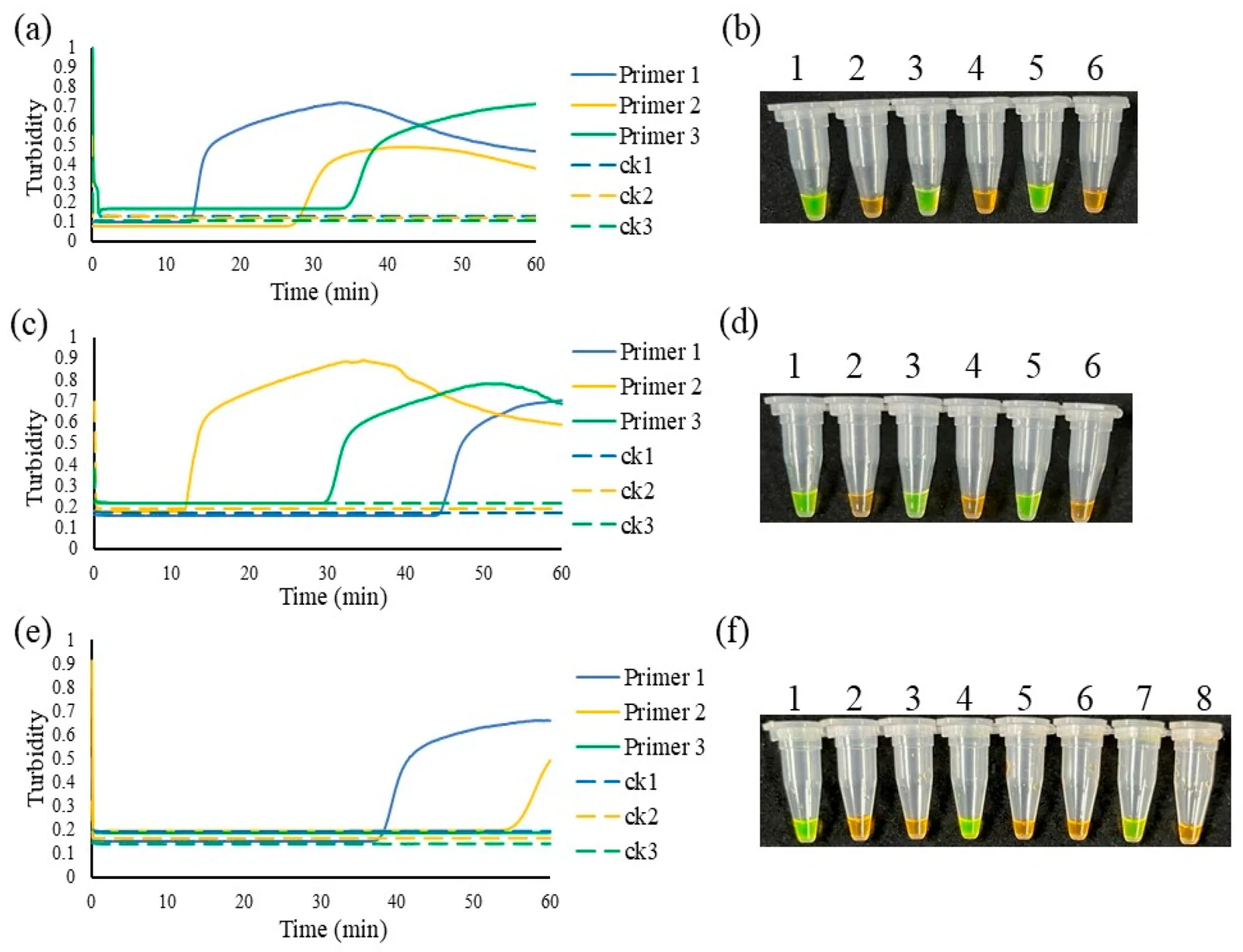
Optimal LAMP primer screening for APYLaOMV, APV1 and APLTV1. (**a**,**c**,**e**) Amplification curve. (**b**,**d**,**f**) Visual detection. In panel (**b**) 1: APYLaOMV-P1; 3: APYLaOMV-P2; 5: APYLaOMV-P3; 2, 4, 6: ck1, ck2,ck3 (blank control). In panel (**d**) 1: APV1-P1; 3: APV1-P2; 5: APV1-P3; 2, 4, 6: ck1, ck2, ck3 (blank control). In panel (**f**), 1: APLTV1-P1; 4: APLTV1-P2; 7: APLTV1-P3; 2, 3, 5, 6, 8: all blank controls.

According to the amplification curves and visual detection results shown in Figure 1, the optimal primer set for each virus was determined as follows: For APYLaOMV, primer set 1 (APYLaOMV-P1) exhibited the earliest peak appearance, high amplification efficiency and a positive color reaction and was therefore selected for subsequent experiments. For APV1, primer set 2 (APV1-P2) produced the earliest peak with high amplification efficiency and a positive color reaction, and was therefore selected. For APLTV1, primer set 1 (APLTV1-P1) showed the earliest peak, primer set 2 showed a delayed peak, and no peak was observed for primer set 3; accordingly, APLTV1-P1 was the only set that satisfied all four criteria: earliest time to amplification peak, highest amplification efficiency, positive color change after SYBR Green I staining, and reproducibility across three independent replicates, and was therefore unambiguously selected as the optimal primer set and was unambiguously selected as the optimal primer set. As shown in Figure 1, the turbidity curves of some positive reactions showed a slight decrease after the peak. This does not affect the interpretation of the results, because positivity is judged by the presence of a typical S-shaped amplification curve together with the color change after SYBR Green I staining, rather than by the trend of the plateau phase.

### 2.2. Amplification Results of the Three Viruses at the Optimized LAMP Temperatures

After the optimal primer sets had been selected, the amplification temperature of each assay was optimized to further improve the efficiency and specificity of the reactions. In the primer-screening experiments described above (Figure S1), all reactions were performed at 65 °C; therefore, temperature gradients covering a range around this initial temperature (59, 61, 62, 63, 65, 67, 68 °C) were tested. To ensure comparability with the primer-screening results, the same batch of cDNA templates (reverse-transcribed from RT-PCR-confirmed APYLaOMV-, APV1-, and APLTV1-infected areca palm leaves) and the corresponding optimal primer sets (APYLaOMV-P1, APV1-P2, and APLTV1-P1) were used throughout the optimization. As shown in the amplification curves and color development results in Figure S1, the optimal reaction temperatures varied among the three viruses. For APYLaOMV and APV1, the earliest peak emergence and highest amplification efficiency were observed at 65 °C. By contrast, the optimal reaction temperature for APLTV1 was 63 °C, at which the earliest peak and the highest amplification efficiency were achieved.

### 2.3. Optimization of Mg^2+^ Concentration for LAMP Assay of the Three Viruses

Because magnesium ions serve as a cofactor for the Bst DNA polymerase and the concentration of Mg^2+^ directly affects the efficiency and specificity of LAMP amplification, the Mg^2+^ concentration in each optimized reaction system was further optimized. In the experiments described above, Mg^2+^ was used at 8 mM, the default concentration recommended by the manufacturer of the Bst DNA polymerase and commonly adopted in published LAMP protocols. To identify the optimal concentration for each virus, a gradient of 4, 6, 8, 10, 12, and 14 mM Mg^2+^ was tested, covering a range that brackets the default value: the lower bound (4 mM) was chosen to test whether suboptimal cofactor levels would compromise amplification, and the upper bound (14 mM) was chosen to test whether excess Mg^2+^ would improve efficiency or, alternatively, promote non-specific amplification. All reactions were performed using the optimal primer sets (APYLaOMV-P1, APV1-P2, and APLTV1-P1) and the previously optimized reaction temperatures (65 °C for APYLaOMV and APV1; 63 °C for APLTV1), with the same batch of cDNA templates used in the primer-screening and temperature-optimization experiments. As shown in Figure S2, no amplification curve was observed for APYLaOMV at 4 mM Mg^2+^, indicating that this concentration was insufficient for efficient amplification. At 12 and 14 mM Mg^2+^, this LAMP reaction showed early peak emergence and high amplification efficiency. To reduce reagent costs without compromising assay performance, 12 mM was selected as the optimal Mg^2+^ concentration for APYLaOMV. For APV1 and APLTV1, the earliest peak appearance and the highest amplification efficiency were achieved at 10 mM Mg^2+^; therefore, 10 mM was determined as the optimal Mg^2+^ concentration for both viruses.

### 2.4. Optimization of dNTPs Concentration for LAMP Assay of the Three Viruses

The dNTP concentration influences both the rate of amplification and the fidelity of the reaction and was therefore optimized for each virus. The gradients were as follows: 0.5, 0.8, 1.1, 1.4, 1.7 and 2.0 mM for APYLaOMV, and 0.4, 0.6, 0.8, 1.0, 1.2 and 1.4 mM for APV1 and APLTV1. All reactions were performed with the optimal primer sets, the optimized Mg^2+^ concentrations and the optimized temperatures, using the same batch of cDNA templates. As shown in Figure S3, the earliest peak appearance and the highest amplification efficiency were obtained at 1.7 mM for APYLaOMV, 1.0 mM for APV1 and 0.8 mM for APLTV1; these concentrations were therefore selected as optimal for the respective assays.

### 2.5. Optimization of LAMP Primer Concentration Ratios for the Three Viruses

The ratio of inner primers (FIP/BIP) to outer primers (F3/B3) affects the kinetics of the LAMP reaction and was optimized for each virus. Throughout this manuscript, the primer ratio is expressed as the inner-to-outer ratio (FIP/BIP:F3/B3). The ratio used in the preceding experiments was 1:1, and the following ratios were tested: 1:1, 2:1, 4:1, 8:1 and 16:1 for APYLaOMV and APLTV1, and 2:1, 4:1, 8:1, 16:1 and 24:1 for APV1. All reactions were performed with the optimized conditions determined above and the same batch of cDNA templates.

As shown in Figure S4, an inner-to-outer ratio of 2:1 gave the earliest peak and the highest amplification efficiency for APYLaOMV; For APV1, the 4:1 ratio produced the shortest time to peak and the highest amplification efficiency among the ratios tested; For APLTV1, the 8:1 ratio performed best. These ratios were therefore selected as optimal for the three assays. The divergent optimal ratios are likely to reflect differences in the relative binding efficiencies of the inner and outer primers for the three target sequences.

### 2.6. Sensitivity of the LAMP Assays for the Three Viruses

**Figure 2:**
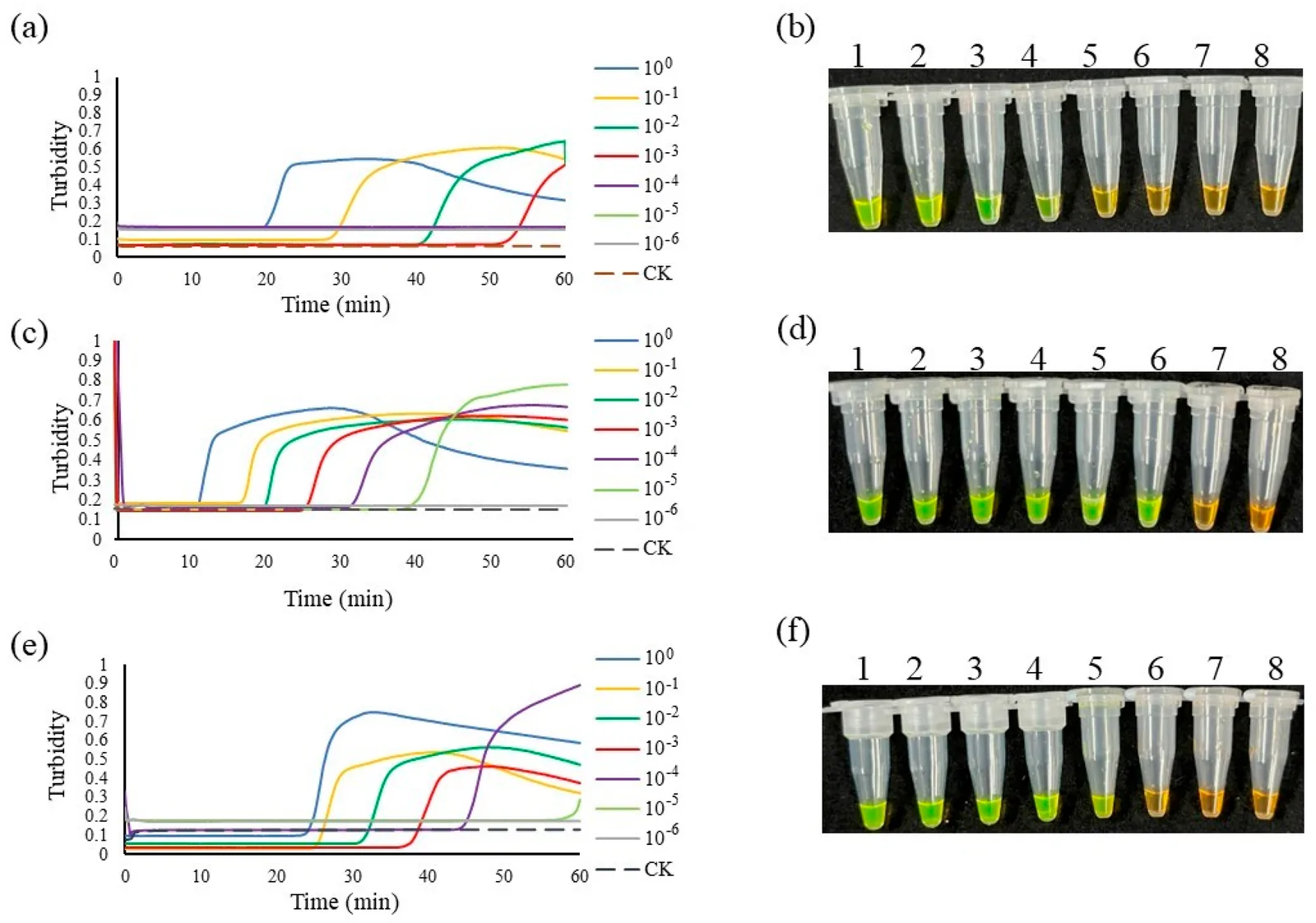
Sensitivity test of LAMP assay for APYLaOMV, APV1 and APLTV1. (**a**,**c**,**e**) Amplification curve. (**b**,**d**,**f**) Visual detection. In panel (**b**) for APYLaOMV, 1: 10^0^; 2: 10^−1^; 3: 10^−2^; 4: 10^−3^; 5: 10^−4^; 6: 10^−5^; 7: 10^−6^; 8: ck (blank control). In panel (**d**) for APV1, 1: 10^0^; 2: 10^−1^; 3: 10^−2^; 4: 10^−3^; 5: 10^−4^; 6: 10^−5^; 7: 10^−6^; 8: ck (blank control). In panel (**f**) for APLTV1, 1: 10^0^; 2: 10^−1^; 3: 10^−2^; 4: 10^−3^; 5: 10^−4^; 6: 10^−5^; 7: 10^−6^; 8: ck (blank control).

The aim of this experiment was to determine the lowest amount of target that each assay can detect. To allow the three assays to be compared with one another and with other methods, the detection limits were determined against a quantified standard. The cDNAs of the three viruses were serially diluted in 10-fold increments and used as templates for LAMP amplification. The results (Figure 2) revealed that the amplification curve of APYLaOMV was detectable down to a dilution of 10^−3^, indicating a detection limit of 10^−3^. For APV1, the curve was observed at a dilution of 10^−5^; thus, its detection limit was determined to be 10^−5^. Although an amplification curve was obtained for APLTV1 at the 10^−5^ dilution, no valid Tt value could be generated for this curve; consequently, the detection limit of APLTV1 was defined as 10^−4^ dilution.

### 2.7. Specificity Analysis of LAMP Assays for the Three Viruses

**Figure 3:**
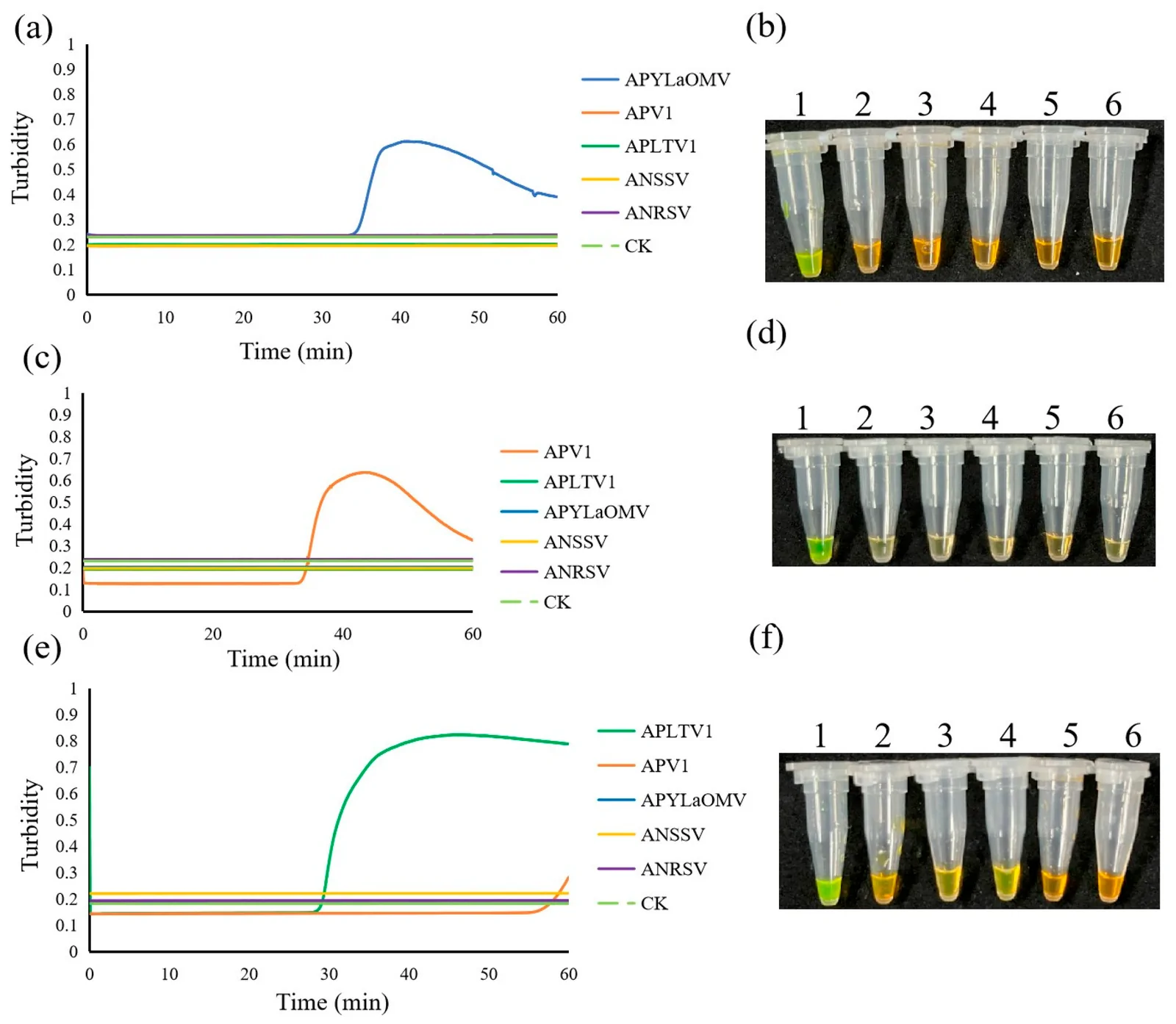
Specificity test of the LAMP assay for APYLaOMV, APV1 and APLTV1. (**a**,**c**,**e**) Amplification curve. (**b**,**d**,**f**) Visual detection. In panel (**b**) for APYLaOMV, 1: APYLaOMV; 2: APV1; 3: APLTV1; 4: ANSSV; 5: ANRSV; 6: ck (blank control). In panel (**d**) for APV1, 1: APV1; 2: APYLaOMV; 3: APLTV1; 4: ANSSV; 5: ANRSV; 6: ck (blank control). In panel (**d**) for APLTV1, 1: APLTV1; 2: APV1; 3: APYLaOMV; 4: ANSSV; 5: ANRSV; 6: ck (blank control).

To evaluate the specificity of the LAMP assays, cross-reactivity tests were performed using cDNAs of the three target viruses (APYLaOMV, APV1, and APLTV1) and two non-target areca palm viruses (ANSSV and ANRSV). Each reaction was carried out independently in a separate tube, with only one viral cDNA used as the template per reaction; a no-template control (NTC) was included in each run to monitor contamination. Specifically, the LAMP assay optimized for APYLaOMV was tested against cDNAs of APYLaOMV, APV1, APLTV1, ANSSV, and ANRSV in parallel, and the same set of templates was applied to the assays optimized for APV1 and APLTV1, respectively. The results showed that for each of the three assays, amplification curves and distinct positive color reactions were observed only when the corresponding target virus cDNA served as the template, whereas no amplification or color change was detected with any of the non-target viral cDNAs or in the NTC (Figure 3). These results demonstrated that the established LAMP methods possessed good specificity for the three target viruses.

### 2.8. Application of the LAMP Detection Method

The established LAMP assay was compared with RT-PCR using a total of 26 samples collected from different regions of Hainan Province for the detection of three viruses. The results showed that the total number of positive detections by LAMP was higher than that by RT-PCR, which was consistent with its lower limit of detection. For APYLaOMV, the detection rate of RT-PCR (84.6%, 22/26) was marginally higher than that of LAMP (80.8%, 21/26) (Table 1).

## 3. Discussion

In this study, three independent two-step RT-LAMP assays were developed and optimized for the detection of APV1, APLTV1, and APYLaOMV, three areca palm viruses that are difficult to distinguish by symptoms alone. Each assay was designed, optimized, and validated separately; the three assays are intended to be used as parallel tools rather than as a multiplex format.

Primer design and target selection. LAMP primers were designed on regions that were conserved within each virus (Section 2.1). For APV1 and APLTV1, all available complete CP gene sequences were aligned separately for each virus with DNAMAN 8, and conserved stretches of the CP gene were used for primer design; for APYLaOMV, only a single complete genome sequence is currently available (GenBank OR204700), so primers were designed directly on conserved sub-regions of that genome. The selected primer sets (APV1-P2, APLTV1-P1 and APYLaOMV-P1) each produced the earliest amplification peak among the three candidates tested and satisfied all four selection criteria (Figure 1). The fact that different target regions were used for the three viruses (the CP gene for APV1 and APLTV1 and the complete genome for APYLaOMV) reflects the availability of sequence data and the need to identify, for each virus, a region that is both sufficiently conserved and virus-specific; it does not indicate that a region conserved among the three viruses was sought.

Optimization of reaction conditions. The three assays showed distinct optimal conditions (Figures S1–S4): the optimal temperature was 65 °C for APV1 and APYLaOMV and 63 °C for APLTV1; the optimal Mg^2+^ concentration was 12 mM for APYLaOMV and 10 mM for APV1 and APLTV1; the optimal dNTP concentration was 1.7 mM for APYLaOMV, 1.0 mM for APV1 and 0.8 mM for APLTV1; and the optimal inner-to-outer primer ratio was 2:1 for APYLaOMV, 4:1 for APV1 and 8:1 for APLTV1. The lower optimal temperature of the APLTV1 assay is consistent with the lower melting temperatures of its primer set, which reduce primer-template stability at higher temperatures. The higher Mg^2+^ requirement of APYLaOMV may reflect the base composition of its target region, because Mg^2+^ stabilizes primer-template duplexes in a sequence-dependent manner. The divergent optimal primer ratios probably reflect differences in the relative binding efficiencies of the inner and outer primers across the three target sequences. Taken together, these results underscore the need for empirical optimization of every LAMP assay rather than the application of a single universal set of conditions.

Analytical sensitivity. The sensitivity of the established LAMP assays was evaluated using 10-fold serial dilutions of viral cDNAs (Figure 2). The detection limits were 10^−3^ dilution for APYLaOMV, 10^−5^ for APV1, and 10^−4^ for APLTV1. These detection limits are comparable to or exceed those of conventional RT-PCR methods routinely used for these viruses [12,13,16], demonstrating the high analytical sensitivity of the LAMP platform. The apparent differences among the three assays should be interpreted with caution, for two reasons. First, the three viruses differ in genome type: APV1 and APYLaOMV have (+)ssRNA genomes, whereas APLTV1 has a dsRNA genome. dsRNA is more difficult to denature and reverse-transcribe than ssRNA, so less APLTV1 cDNA is recovered per unit of tissue than for the two ssRNA viruses; part of the difference in the apparent detection limit therefore reflects the extraction and reverse-transcription steps rather than the LAMP reaction itself. Second, the amounts of virus accumulated in planta differ among the three viruses. Because the templates were cDNAs reverse-transcribed from total RNA of naturally infected field samples, the initial viral titers of the three preparations were unknown, and the detection limits are therefore expressed as dilution endpoints of the respective cDNA preparations. The differences in detection limits among the three viruses are attributed to differences in the initial cDNA concentrations, primer binding efficiencies, and target sequence accessibility. Determination of the analytical sensitivity using quantified standards (e.g., in vitro-transcribed RNA) will be an important direction for future work.

Specificity and performance on field samples. Under the optimized conditions, none of the three assays amplified the cDNAs of the non-target areca palm viruses ANSSV and ANRSV, or those of the two other target viruses, and no amplification was observed in the no-template controls (Figure 3). The specificity of LAMP is conferred by the use of four to six primers that recognize six to eight distinct regions of the target sequence, which makes non-specific amplification unlikely. This is particularly important for field application, where the three viruses frequently occur as mixed infections and a false-positive result would be misinterpreted as a co-infection. The three assays were validated on 26 field samples. RT-LAMP detected more positive samples than RT-PCR for APV1 and APLTV1. For APYLaOMV, the RT-PCR detection rate reached 84.6%, whereas LAMP achieved 80.8% (Table 1). This single-virus discrepancy corresponds to only one sample (3.8%) and is most likely attributable to the stochastic distribution of viral titers in field samples relative to the limits of detection (LODs) of the two assays: detection efficiencies are not linearly correlated near the LODs, and samples with template concentrations falling in the “gray zone” between the two methods may be detected by one assay but missed by the other. With only 26 samples, a 3.8% difference is within the expected range of random variation and does not undermine the overall higher sensitivity of LAMP across the three viruses. Overall, the LAMP assay performed stably. Negative controls were included in every run to rule out aerosol contamination and ensure result accuracy.

Limitations and future perspectives. Three limitations of the present study should be acknowledged. First, the assays were validated mainly on samples with single infections. Although the specificity of each assay was confirmed against the other viruses and simulated mixed templates were tested, the performance of the assays on naturally co-infected field samples remains to be evaluated in a larger survey. Second, the sample size used for field validation (26 samples) was limited; larger-scale surveys across diverse regions and seasons are required to confirm robustness. Third, the vectors and transmission mechanisms of APLTV1 and APYLaOMV remain unknown; although this is outside the scope of a detection-focused study, it is essential for integrated disease management.

Future work on multiplex detection. It should be emphasized that the three assays reported here are independent, single-target assays and are not intended for multiplexing. Both of the readouts used, turbidity and SYBR Green I staining, are generic indicators of the accumulation of double-stranded DNA and cannot discriminate between amplicons generated from different targets in the same reaction. They are therefore intrinsically unsuitable for true multiplex detection. Multiplex detection of the three viruses in a single tube would require a target-specific readout, for example (i) sequence-specific fluorescent probes with distinct emission spectra, (ii) lateral-flow strip detection using differentially labelled primers, or (iii) a microfluidic device with parallel reaction chambers. Future work in our laboratory will explore these strategies.

In conclusion, three independent RT-LAMP assays were established for the detection of APV1, APLTV1 and APYLaOMV. The assays are specific, their analytical sensitivity is comparable to or better than that of conventional RT-PCR, and allow visual readout of the results. They provide practical tools for routine virus identification, are particularly useful when YLD incidence increases, and are well adapted to remote plantations where laboratory infrastructure is limited. These findings provide a technical foundation for the quarantine and integrated management of YLD in areca palm production.

## 4. Materials and Methods

### 4.1. Plant Materials

Healthy and infected leaves were collected from areca palm-growing regions of various cities and counties on Hainan Island. Positive samples of APV1, APLTV1, or APYLaOMV, identified via RT-PCR and sequencing, were used to establish and optimize the LAMP assays, with virus-free healthy samples serving as negative controls. Subsequently, field samples of areca palms were collected from plantations; a total of 26 samples were collected from 26 different regions of Hainan, immediately frozen in liquid nitrogen, and transported to the laboratory for further analysis. These confirmation samples included those with single infections (APV1, APLTV1, or APYLaOMV); double and triple mixed infections of the three viruses were not included in this study.

### 4.2. RNA Extraction and RT-PCR

Total RNA was isolated from areca leaf samples as previously described [9]. First-strand cDNA was synthesized from 5 µg total RNA using a RevertAid First Strand cDNA Synthesis Kit (#K1622, Thermo Fisher Scientific, Shanghai, China) in a 20 µL reaction mixture with random primers, according to the manufacturer’s protocol.

To compare the LAMP results with those obtained by RT-PCR, RT-PCR was performed under the following conditions. The RT-PCR primers for the three viruses are listed in Table 2. RT-PCR amplification was performed in a 25 μL reaction volume containing 12.5 μL of 2× PCR Master Mix, 0.5 μL of each primer (10 μM), 1 μL of cDNA template, and 10.5 μL of nuclease-free water. The amplification was carried out in a T100 Thermal Cycler (Bio-Rad, Hercules, CA, USA) under the following conditions: initial denaturation at 94 °C for 5 min, followed by 35 cycles of denaturation at 94 °C for 30 s, annealing at 52–58 °C for 30 s, and extension at 72 °C for 1 min, with a final extension at 72 °C for 10 min. The PCR products 5 μL were separated by electrophoresis on a 1.5% agarose gel in 1× TAE buffer, stained with GelRed, and visualized under UV light. A DL2000 DNA marker (TaKaRa, Dalian, China) was used to estimate the size of the amplicons. PCR-grade water and cDNA from sequencing-confirmed virus-infected samples served as the negative and positive controls, respectively.

### 4.3. Design and Synthesis of LAMP Primers for the Three Viruses

LAMP primer sets were designed using the online primer design software PrimerExplorer V5.0 (http://primerexplorer.jp/lampv5e/index.html, accessed on 16 July 2023) and synthesized by Sangon Biotech (Shanghai) Co., Ltd. (Shanghai, China), as shown in Table 3. The target nucleotide sequences utilized in this study included the spliced whole-genome sequence of APYLaOMV (Accession No. OR204700), the CP gene sequence of APLTV1 isolate TCWP531 (Accession No. PX244090.1), and the CP gene sequence of APV1 isolate QZWL1 (Accession No. MN296265.1). All acquired sequences were aligned using DNAMAN 8 software to determine the final amplified target regions. Each LAMP primer set comprised a forward outer primer (F3), a reverse outer primer (B3), a forward inner primer (FIP, consisting of F1c + F2), and a reverse inner primer (BIP, consisting of B1c + B2).

### 4.4. Optimization of LAMP Reaction System and Conditions

The initial LAMP reaction system was prepared to a total volume of 20 µL, containing 10× Bst 2.0 (Novorprotein, Suzhou, China, 8 U/µL) Buffer with Mg^2+^ (2.0 µL), MgSO_4_ (100 mM, 0.8 µL), FIP/BIP (40 µM, 0.8 µL), F3/B3 (10 µM, 0.8 µL), Bst 2.0 DNA Polymerase (0.8 µL), cDNA (2.0 µL), dNTP mix (10 mM) and deionized water to a final volume of 20 µL. The mixture was incubated at 65 °C for 1 h in an LA-500C real-time turbidimeter (Eiken Chemical Co., Ltd., Tokyo, Japan), followed by inactivation at 85 °C for 5 min. After the reaction, 1.0 µL of SYBR Green I nucleic acid stain was added to the products. The amplification results were evaluated based on the amplification curves recorded by the turbidimeter and the color changes in the products after staining. A positive control (P+) and a negative control (N−) from a LAMP kit (Eiken Chemical Co., Ltd.) were used as experimental controls, and each experiment was repeated three times.

#### 4.4.1. Evaluation of LAMP Amplification Products

Two approaches were used to assess the results of the LAMP assay. In the first method, reaction tubes were placed in a real-time constant-temperature fluorescence detector to monitor fluorescence signals continuously. An S-shaped amplification curve indicated a positive result, confirming the presence of the target virus in the sample; conversely, the absence of an S-shaped curve was defined as negative. For the second approach, fluorescent visual detection reagent (SYBR Green I) was added after incubation. The results were judged by color change: an emerald green color of the reaction mixture indicated a positive result, while an orange color indicated a negative result. It should be noted that the turbidimeter monitors the real-time increase in turbidity caused by the accumulation of magnesium pyrophosphate precipitate during amplification, whereas SYBR Green I staining of the amplicons provides the visual colorimetric readout.

#### 4.4.2. Screening of LAMP Primers for APYLaOMV, APLTV1, and APV1

Using the cDNAs of APYLaOMV, APLTV1, and APV1 as templates, three sets of LAMP primers were designed based on their respective target sequences for LAMP amplification. The optimal primer set was selected according to the peak appearance time.

#### 4.4.3. Optimization of LAMP Amplification Temperature for the Three Viruses

LAMP amplification of APYLaOMV, the reaction temperatures were set at 59, 62, 65, and 68 °C. For APV1 and APLTV1, the reaction was performed at 59, 61, 63, 65, and 67 °C. The temperature yielding the earliest peak emergence was selected as the optimal reaction temperature.

#### 4.4.4. Optimization of Mg^2+^ Concentration for APYLaOMV, APLTV1, and APV1 LAMP Assays

The amount of MgSO_4_ was adjusted to set the Mg^2+^ concentration in the reaction system at 4, 6, 8, 10, 12, and 14 mM for LAMP amplification. The optimal Mg^2+^ concentration was selected based on peak emergence time of amplification curves and reagent cost.

#### 4.4.5. Optimization of dNTPs Concentration in the LAMP Assay for the Three Viruses

The amount of dNTPs was adjusted to set the concentrations at 0.5, 0.8, 1.1, 1.4, 1.7, and 2.0 mM for LAMP amplification of APYLaOMV. For APV1 and APLTV1, dNTP concentrations were set to 0.4, 0.6, 0.8, 1.0, 1.2, and 1.4 mM, respectively. The optimal dNTP concentration was selected based on peak emergence time.

#### 4.4.6. Optimization of LAMP Primer Concentration Ratio for APYLaOMV, APLTV1, and APV1

The concentration ratios of inner primers (FIP/BIP) to outer primers (F3/B3) were set at 2:1, 4:1, 8:1, 16:1, and 24:1 for LAMP amplification of APV1. For APYLaOMV and APLTV1, the ratios were adjusted to 1:1, 2:1, 4:1, 8:1, and 16:1, respectively. The optimal primer ratio was determined according to the peak emergence time.

#### 4.4.7. Sensitivity and Specificity Analysis of the Three Viruses LAMP Assays

The cDNAs of APV1, APYLaOMV, and APLTV1 were serially diluted 10-fold and used as templates for LAMP amplification. The highest dilution factor that still generated an amplification curve was defined as the detection limit. For specificity testing, LAMP amplification was performed using the cDNAs of APV1, APLTV1, APYLaOMV, ANSSV, and ANRSV as templates. Positive signals were expected only for the target virus in each assay, confirming the specificity of the LAMP method.

## Figures and Tables

**Table 1:** RT-PCR and LAMP Detection of Three Areca palm viruses in Hainan.

Virus Species	APYLaOMV		APV1		APLTV1	
Sample No.	RT-PCR	RT-LAMP	RT-PCR	RT-LAMP	RT-PCR	RT-LAMP
LG3	+	+	+	−	−	+
LG4	+	+	+	+	−	−
LD3-4	+	+	+	+	−	+
WC1-4	+	+	+	+	−	−
SL3-7	−	−	−	−	−	−
PL1-4	+	+	+	+	−	+
QY2-1	+	+	+	+	+	−
BY4-2	−	−	−	−	−	−
XZ1-5	+	+	+	+	+	−
TM1-5	+	+	+	+	+	+
LMS3-4	+	+	+	+	+	+
NK4-4	+	+	−	+	+	+
XZ1-6	+	+	+	+	+	−
QJ4-1	−	−	−	−	−	−
SD3-2	+	+	+	+	+	+
DF1-4	+	+	−	+	+	+
SL3-2	+	+	+	+	+	+
DL2-5	+	+	−	+	−	+
NF1-5	+	+	−	+	−	+
HL3-5	+	+	+	+	+	+
YL5-3	+	+	−	−	−	+
HK25	+	−	+	−	−	−
HZ5-5	+	+	+	+	+	+
HKHQ	−	−	+	+	−	−
DZP1-1	+	+	+	+	+	−
YZ2-1	+	+	+	+	+	+
Detection rate	84.6%	80.8%	69.2%	76.9%	50.0%	57.7%

Note: “+” means positive result; “−” means negative result.

**Table 2:** RT-PCR primers for the three viruses.

Primer Name	Primer Sequence (5′-3′)	Tm (°C)	Expected Size (bp)
APYLaOMV-630F	GAATTACCCACGCCTCCAG	53.2	630
APYLaOMV-630R	GAGCTTTCCAGGACATGCC	53.2	
APV1-F	CCACTCTTCTGGTAGTATCAAGG	57.1	1000
APV1-R	CAGAAGCATAAGATTGTGACATTTTTACCG	67.4	
APLTV1-F	TAGCTCAATGGCGTAGGCG	62.8	1500
APLTV1-R	CCCGCTTCTCTAGGTTGTGC	62.1	

**Table 3:** Primer sequences used in LAMP for APYLaOMV, APV1 and APLTV1.

Primer Group	Primer Name	Primer Sequence (5′-3′)
APYLaOMV-P1	P7-1-F3	CATTACACAGACAATTGAGGAG
	P7-1-B3	CGCGTATTTCATTCTTTGATCC
	P7-1-FIP	GGTAGAATTCCCATCCCCAGTGATTACCGTTCAGGTATTCATCG
	P7-1-BIP	TAAACCATTCCTATTTGGAGGCTGGGTGAAATACCGGAGAGAAG
APYLaOMV-P2	P7-2-F3	ATATCAACCCTGTAGGAAAGT
	P7-2-B3	CCGTTCTTGTAAATTTCTTCAAG
	P7-2-FIP	GACTGGAGGCGTGGGTAATTATTTACAGCATTCAATGATGACT
	P7-2-BIP	GGACCAGAACGTGTGGGATATGAAACCATCACGGCATAC
APYLaOMV-P3	P7-3-F3	AGGAGATTACCGTTCAGGTA
	P7-3-B3	TTGACGGTCATATTCGCG
	P7-3-FIP	CCAGCCTCCAAATAGGAATGGTTTACGCAATTGAACAAGTAATTCG
	P7-3-BIP	GGCTTAGATTCTTCTCTCCGGTTTAAAGCCGGTTACAGCT
APV1-P1	APV1-1-F3	CGATACACACAACAGTTTCA
	APV1-1-B3	CATTCTTTGCAGCTAATCGA
	APV1-1-FIP	GCCTTAAGTGCATTGACCGAATTTAAGTAGATAATAGGTCTTATGATTGG
	APV1-1-BIP	CAACGTGTTGCGAAGGTATGGCCTGATGGTACATAATTAATCTCA
APV1-P2	APV1-2-F3	AGATTAATTATGTACCATCAGGTC
	APV1-2-B3	TGCACAATTGTTTTACCCAT
	APV1-2-FIP	CGACGGAGTGAAATCTGTGATATTTGATTAGCTGCAAAGAATGGATC
	APV1-2-BIP	TTCTCTACTGCAGAAGAACAAAGTTCCACTTTTCGTGATCGCATA
APV1-P3	APV1-3-F3	AGGAGATTACCGTTCAGGTA
	APV1-3-B3	CGAATTTATATAATCCCAAAGAGCT
	APV1-3-FIP	GCTCAGAAATGGAAATGCATAATGAAAATGAATTGTCAACTAAGATGTGG
	APV1-3-BIP	ATGGACGTCGAAAACTAACGCTTTGAGTCCAATCATAAGACCTA
APLTV1-P1	APLTV1-1-F3	GGCGATCTAAATTGCGATT
	APLTV1-1-B3	GCGTTACATACTGTTTAATTGC
	APLTV1-1-FIP	CTAGCCACGCATCTAAATTAGGAATTTCTCTACACCAAAATTAGTAGAGG
	APLTV1-1-F3	CAGAGCCAGTACCAGGTGAGCCAGCAAATATCAGAAGTAGG
APLTV1-P2	APLTV1-2-F3	GGCATGTTTGACTGGTCTA
	APLTV1-2-B3	ATTCGCATAAATATAAACCCCT
	APLTV1-2-FIP	TCCATCTCCGATGTGTATAGATCTTGCTATTCGTAGTATACGAAAGG
	APLTV1-2-BIP	CAAACGAGTAATTTCTATTCAGCCGATACCAGGTAATGGGACTTCA
APLTV1-P3	APLTV1-3-F3	CCGAAGACTTATCTTACCCAA
	APLTV1-3-B3	ATCATAGCGTCAGCTCGA
	APLTV1-3-FIP	CCTGATTCTGAGTCATACCTCTCAGATATTTAAGTGAGAGTGTACCTACC
	APLTV1-3-BIP	AGGTTCACGTTACTTATGCTAGCTATCCTCTACTAATTTTGGTGTAG

## Data Availability

All data used in this study are publicly available on NCBI. The CP gene sequences obtained in this study have been deposited in GenBank under the accession numbers PX243804–PX244167.

## Supplementary Materials

The following supporting information can be downloaded at: https://www.mdpi.com/article/10.3390/plants15203234/s1.
