# Supplementary material for: Development of Loop-Mediated Isothermal Amplification Assays for Rapid and Sensitive Detection of Three Viruses Infecting Areca catechu L
Source: Plants (Basel). 2026 Oct 21;15(20):3234. doi: 10.3390/plants15203234 (PMC13708926; doi:10.3390/plants15203234)
Supplement: Supplementary file 1 [file plants-15-03234-s001.zip › plants-4548389-supplementary.pdf]

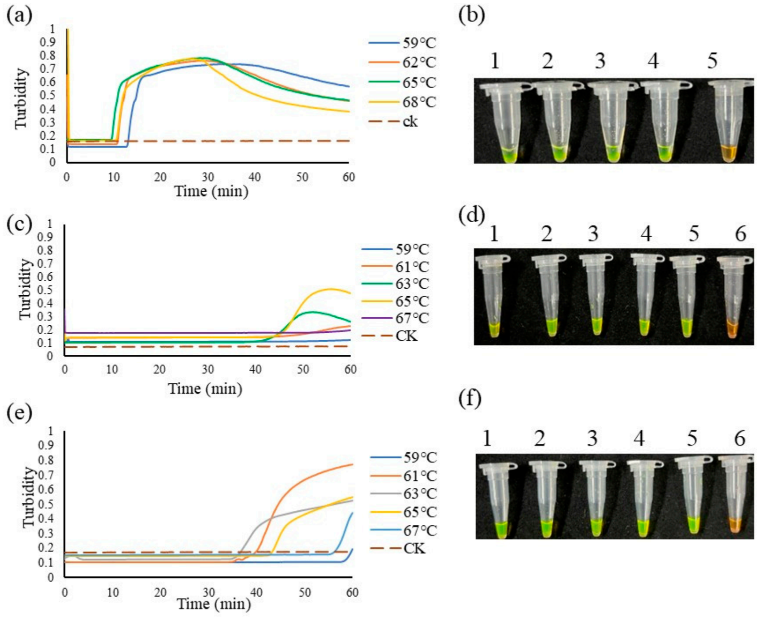

Figure S1. Temperature optimization of LAMP assay for APYLaOMV1, APV1 and APLTV1. a,c,e: Amplification curve. b,d,f:Visual detection. In panel b for APYLaOMV1 ,1: 59℃; 2: 62℃; 3: 65℃; 4: 68℃ ; 5: ck (blank control). In panel d for APV1,1: 59℃; 2: 61℃; 3: 63℃; 4: 65℃; 5: 67℃; 6: ck (blank control). In panel f for APLTV1,1: 59°C; 2: 61°C; 3: 63°C; 4: 65°C; 5: 67°C; 6:ck (blank control).

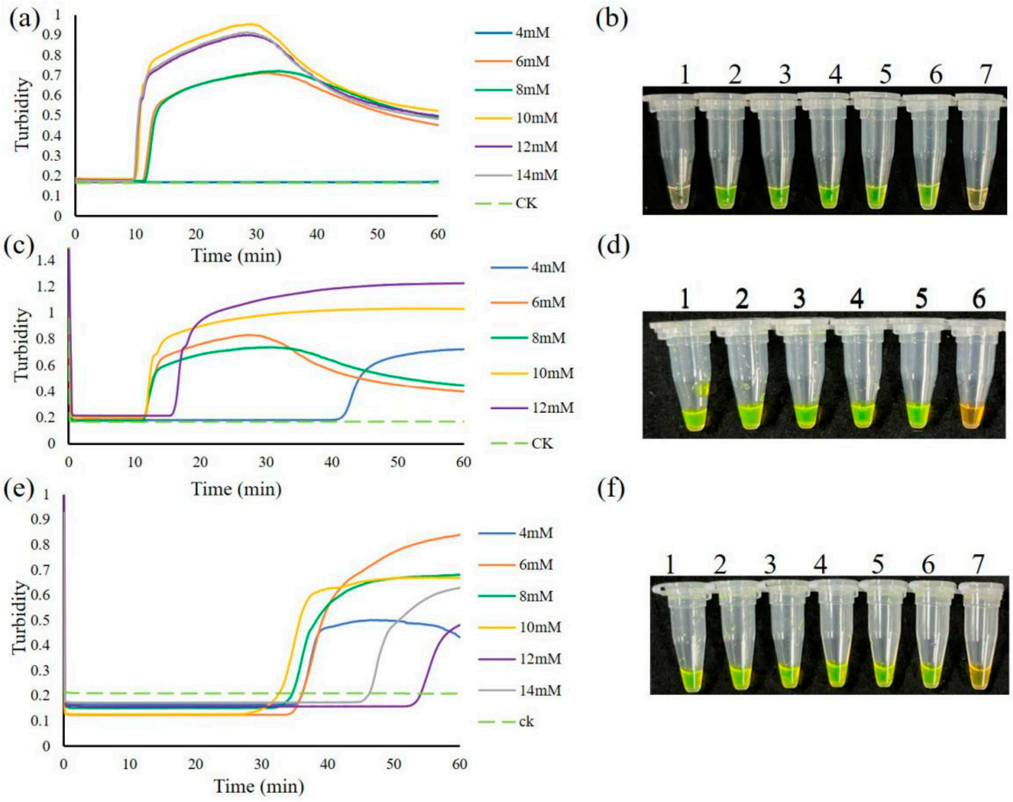

Figure S2. Mg^{2+} concentration optimization of RT-LAMP assay for APYLaOMV,APV1 andAPLTV1. a, c,e: Amplification curve. b,d,f: Visual detection. In panel b for APYLaOMV,1: 4 mM; 2: 6 mM; 3: 8 mM; 4: 10 mM; 5: 12 mM; 6: 14 mM; 7: ck(blank control).In panel d for APV1,1: 4 mM; 2: 6 mM; 3: 8 mM; 4: 10

mM; 5: 12 mM; 6: 14 mM; 7: ck(blank control). In panel f for APLTV1,1: 4 mM; 2: 6 mM; 3: 8 mM; 4: 10 mM; 5: 12 mM; 6: 14 mM; 7: ck(blank control)

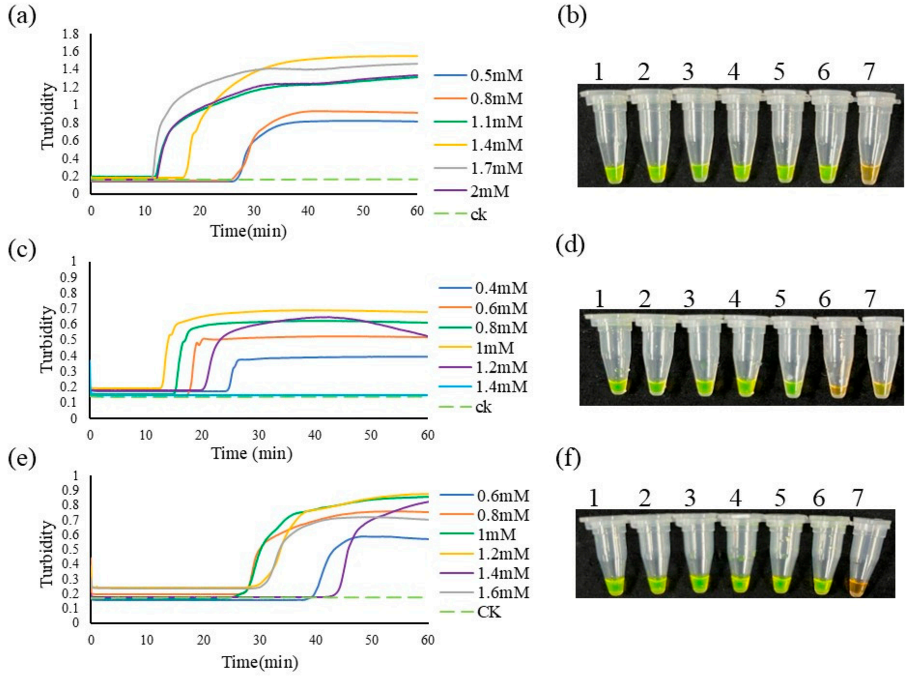

Figure S3. dNTP concentration optimization of LAMP assay for APYLaOMV,APV1 and APLTV1. a,c,e: Amplification curve. b,d,f: Visual detection. In panel b for APYLaOMV，1: 0.5 mM; 2: 0.8 mM; 3: 1.1 mM; 4: 1.4 mM; 5: 1.7 mM; 6: 2.0 mM; 7: ck(blank control).In panel d for APV1,1: 0.4 mM; 2: 0.6 mM; 3: 0.8 mM; 4: 1.0 mM; 5: 1.2 mM; 6: 1.4 mM; 7: ck(blank control).In panel f for APLTV1，1: 0.4 mM; 2: 0.6 mM; 3: 0.8 mM; 4: 1.0 mM; 5: 1.2 mM; 6: 1.4 mM; 7: ck(blank control).

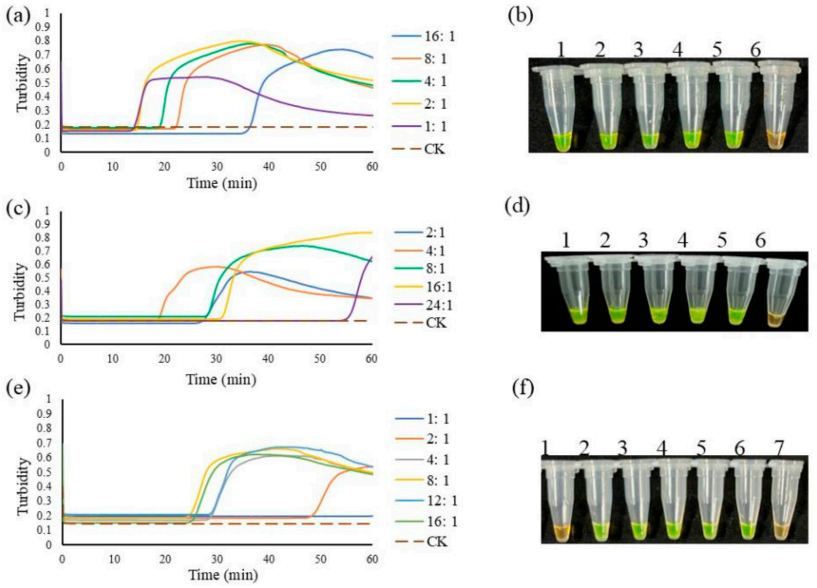

Figure S4. Primer concentration ratio optimization of LAMP assay for APYLaOMV,APV1 and APLTV1. a, c,e: Amplification curve. b,d,f: Visual detection. In panel b for APYLaOMV, 1: 1:1; 2: 2:1; 3: 4:1; 4: 8:1; 5: 16:1; 6: ck(blank control). In panel d for APV1,1: 2:1; 2: 4:1; 3: 8:1; 4: 16:1; 5: 24:1; 6: ck(blank control). In panel f for APLTV1, 1: 1:1; 2: 2:1; 3: 4:1; 4: 8:1; 5: 12:1; 6: 16:1; 7: ck(blank control).
